# Repurchase intentions of new e-commerce users in the COVID-19 context: The mediation role of brand love

**DOI:** 10.3389/fpsyg.2022.968722

**Published:** 2022-08-01

**Authors:** Yi Ding, Ruonan Tu, Yahong Xu, Sung Kyu Park

**Affiliations:** ^1^Department of International Trade, Changwon National University, Changwon, South Korea; ^2^Department of Law, Dong-A University, Busan, South Korea

**Keywords:** brand experience, brand loyalty, repurchase intention, brand attachment, brand love, new e-commerce users

## Abstract

The use of e-commerce has exploded due to the impact of COVID-19. People with no experience in e-commerce prior to the COVID-19 pandemic began online shopping for their safety following the pandemic outbreak. As such, these newly joined customers have played a vital role in the rapid development of e-commerce. Maintaining these customers and increasing their repurchase intention is a core issue for e-commerce platform companies. Thus, using new e-commerce users as the participants, this study investigated the structural relationship between brand experience, brand emotional factors (brand attachment and brand love), brand loyalty, and repurchase intention with brand love as the mediator. Research on the multidimensional brand experience (i.e., sensory, emotional, behavioral, and cognitive) from Chinese customers’ perspective is still lacking, and our study attempts to fill this gap. A structured questionnaire and hypotheses were designed based on studies and survey of 310 respondents from China in this study. The study results show that, first, the four dimensions of brand experience have a significant positive correlation with brand emotion, with brand cognitive experience having the greatest impact on consumer brand emotion. Second, the influence of brand emotion on brand loyalty is positive and significant, and brand attachment has a stronger influence than brand love on brand loyalty. In addition, brand loyalty has a positive effect on repurchase intention. Finally, brand love plays a mediating role on the relationship between brand attachment and brand loyalty. To enhance customers’ brand attachment and love for e-commerce platforms, companies must enhance customers’ interest and curiosity in their products. And companies will improve their services to customers by introducing artificial intelligence algorithms to increase customers’ repurchase intention, which will ultimately increasing their profitability. This study contributes to the development of e-commerce platform companies.

## Introduction

The outbreak of the COVID-19 pandemic in early 2020 has seriously disrupted the functioning of the world economy: countries worldwide took various control measures at the beginning of the outbreak. In China, the quarantine policy was mainly well implemented, because of restrictions on movement, many consumers have shifted from offline to online shopping, presenting a massive opportunity for online e-commerce platforms. Two years into the pandemic, countries worldwide are attempting to recover economically, and restrictions on movement are being gradually lifted; however, the shift in consumer behavior that occurred during the COVID-19 pandemic is expected to persist.

Despite living in the internet society, there are many people who have no experience in online shopping. There are multiple reasons, such as that they do not need to shop online because they live in an environment with complete living facilities, dislike changing their habits, are skeptical about the safety of online shopping, and so on. The outbreak of the COVID-19 pandemic in early 2020 coincided with the Chinese New Year consumption season; compared with investment and exports, the pandemic’s impact on short-term consumption is more significant. In the general environment of the pandemic, e-commerce has become the first choice for people to buy groceries, people with no experience in online shopping may try to access e-commerce platforms and join online shopping. This particular group of people has had a significant positive impact on e-commerce platforms.

During the COVID-19 pandemic, the business landscape has faced rapid transformations during the quarantine period. Ultimately, the COVID-19 crisis accelerated the development of digital commerce (Gu et al., 2021). As a result of the COVID-19 impact, consumers are increasingly turning to online purchases (Thuy Tran, 2021). Their online shopping experience has made them accumulate a level of trust in e-commerce. The prediction is that although some consumers will return to offline shopping after the pandemic, the scope is likely to be focused on goods that require a physical experience. For products regularly purchased daily, e-commerce is still the most convenient way to shop in terms of convenience, habit, and competitive prices. The intensification of competition, the increase in the demand for experience, and the constant changes in the general environment make the e-commerce platform face enormous pressure for survival and development. E-commerce platforms can only continuously optimize and improve the brand experience to make consumers feel good about their brands (Batat, 2019), thus enhancing customer loyalty and reducing customer loss.

To reduce customer churn, it is first necessary to create a memorable brand experience for customers, which is defined as “the behavioral responses that consumers elicit through brand stimuli, including brand design, identity, packaging, communication, and environment, as well as consumers’ subjective and internal reactions” (Brakus et al., 2009, p. 53). This study draws on Brakus’ four-dimensional approach, which classifies explicitly brand experience into four dimensions: sensory, emotional, behavioral, and cognitive. A good brand experience creates a intense emotional attachment to a specific brand. According to Lacuilhe (2000), brand attachment can be seen as a long-term and strong emotional response of consumers to a brand and an expressed psychological closeness. Brand experience through a positive approach can create a sense of connection between the consumer and the brand and facilitate the establishment of an emotional bond (Mahr et al., 2019).

Brand attachment can be transformed into brand love after reaching a certain level of accumulation. The concept of brand love was first introduced by Carroll and Ahuvia (2006). They defined it as a strong emotional attachment of consumers to a specific brand, including enthusiasm, attachment, positive evaluation, positive emotion, and love for the brand. Prior research has shown that brand love mediates the relationship between customer satisfaction and loyalty. Consumers with brand love generate stronger loyalty (Roy et al., 2013).

Although several pioneering researchers have constructed models of satisfaction and loyalty (Hussein, 2018; Kataria and Saini, 2019; Ghorbanzadeh and Rahehagh, 2020a), there is a paucity of studies linking brand experience and emotional factors, with most studies taking brand loyalty as a result (Javed et al., 2021; Kim et al., 2021; Torres et al., 2021). The most critical issue in relationship marketing is the retention rate of customers and repurchase intention (Javed and Wu, 2019).

The main research objective of this study is to explore the structural relationships between the four dimensions of brand experience, brand emotional factors (brand attachment and love), brand loyalty, and repurchase intentions. More specifically, this study aims to (1) verify which dimension of brand experience has the most substantial impact on brand emotional factors, (2) investigate the relationship between brand emotional factors and brand loyalty, (3) explore the relationship between brand loyalty and repurchase intentions, and (4) test whether brand love mediates the relationship between brand attachment and brand loyalty.

This study helps to expand the prior research in three ways. First, unlike the previous studies, this study considers Chinese consumers who are new to online shopping during the COVID-19 pandemic outbreak. Second, few studies have examined the multidimensional brand experiences from the perspective of Chinese customers. In addition, many prior studies have considered brand loyalty as the outcome variable (Ferreira et al., 2019; Quan et al., 2020; Khawaja et al., 2021), and we investigate the relationship between brand loyalty and repurchase intention based on previous studies. Finally, this study clarifies the relationship between brand attachment and brand loyalty by examining the mediating effect of brand love, which serves as a valuable insight for previous literature which has underestimated the mediation role of brand love.

The remainder of the paper is structured as follows. Section “Theoretical background and hypothesis formulation” briefly introduces the theoretical background, formulates the hypotheses, and constructs the model with prior research. Section “Methodology” presents the questionnaire, data collection method, and descriptive statistics sample. Section “Analysis results and hypothesis testing” tests the hypotheses and analyses the results. Section “Discussion” discusses both the theoretical and practical aspects of the findings and explains the limitations of the study and future research directions. The final section summarizes this research.

## Theoretical background and hypothesis formulation

E-commerce refers to trading activities and related service activities on the Internet or electronic transactions, which are electronic and networked in all aspects of traditional business activities. The advent of the Internet era does not make the conventional marketing laws disappear.

Brand experience is defined as “the behavioral responses that consumers elicit through brand stimuli, including brand design, identity, packaging, communication, and environment, as well as consumers’ subjective and internal reactions” (Brakus et al., 2009, p. 53). It is necessary to give customers a unique and unforgettable memory of their brand experience to stand out in today’s competitive e-commerce industry so that customers build a positive emotional relationship with the brand (Wijekoon and Fernando, 2020). The concept of brand experience is crucial for establishing a good connection between consumers and brands. Brakus et al.’s (2009) four-dimensional approach is a widely accepted method (Molinillo et al., 2021; Prentice and Nguyen, 2021; Safeer et al., 2021), which classifies brand experience into four dimensions: sensory experience, emotional experience, cognitive experience, and behavioral experience.

The sensory dimension involves sensations evoked by brand-related stimuli perceived through the senses (Brakus et al., 2009). In the case of brands, sensory brand experiences are based on visual, aural, olfactory, gustatory, and tactile experiences (Barnes et al., 2014). The emotional dimension appeals to the sentiments, emotions, and innermost feelings of a consumer. It can include positive feelings or strong emotions related to a product (Schmitt, 1999; Barnes et al., 2014). The cognitive dimension is linked to thoughts, stimulation of curiosity, and problem-solving in encounters with brand stimuli (Barnes et al., 2014). The behavioral dimension includes experiences in which a customer performs physical actions (Schmitt, 1999; Brakus et al., 2009).

Previous research shows that a good brand experience leads to consumer love for a brand (Chen and Qasim, 2020; Junaid et al., 2020). Practical and positive brand experience leads to brand love (Bıçakcıoğlu et al., 2016). To develop consumers’ brand love, companies must enable consumers to have positive brand attitudes, experiences, and psychological closeness to the brand. Customers can experience a brand through four dimensions: sensory, emotional, behavioral, and cognitive. Different brand experiences have different impacts on customers (Ong et al., 2018; Safeer et al., 2021). In this context, the first hypothesis is formulated in this article:

H1. Brand experience will positively affect brand love.

Hypothesis 1-1 Brand sensory experience will positively affect brand love.

Hypothesis 1-2 Brand emotional experience will positively affect brand love.

Hypothesis 1-3 Brand behavioral experience will positively affect brand love.

Hypothesis 1-4 Brand cognitive experience will positively affect brand love.

Brand attachment refers to an emotional and affective bond between a customer and a specific brand (Thomson et al., 2005, p. 105). It is a solid affective and cognitive tie between a customer and a particular brand, such that the brand is regarded as an extension of the self (Park et al., 2006).

Previous studies have demonstrated that brand experience facilitates brand attachment (Kang et al., 2016; Japutra et al., 2018; Huaman-Ramirez and Merunka, 2019). Consumers positively experience a brand, creating brand memories and a sense of connection with the brand, which facilitates the creation of an emotional bond (Reihani et al., 2019). Brand attachment results from brand experience; therefore, brand experience determines the strength of the relationship between the consumer and brand. These reactions generated by a good experience become positive brand memories, thus creating a connection between the consumer and the brand (Gómez-Suárez and Veloso, 2020). Authors hypothesize that this process applies to e-commerce platform brands. Therefore, this study proposes the second hypothesis:

H2. Brand experience will positively affect brand attachment.

Hypothesis 2-1 Brand sensory experience will positively affect brand attachment.

Hypothesis 2-2 Brand emotional experience will positively affect brand attachment.

Hypothesis 2-3 Brand behavioral experience will positively affect brand attachment.

Hypothesis 2-4 Brand cognitive experience will positively affect brand attachment.

Sternberg (1986) proposed the triangular theory of love, then Shimp and Madden (1988) introduced it to the field of consumer research. Carroll and Ahuvia (2006, p. 81) defined brand love as a strong emotional attachment of consumers to a specific brand, which mainly consists of enthusiasm, attachment, positive evaluation, positive emotion and love for the brand. Brand love mediates the relationship between customer satisfaction and loyalty. Consumers who have brand love generate stronger loyalty than those who do not have brand love.

Consumers must first attach to a brand, then love it, and finally feel that it is irreplaceable (Loureiro et al., 2017). Brand attachment is an element of brand love (Campbell, 2017; Rahmatiyah et al., 2017); however, little research has been conducted to show that brand attachment is an antecedent of brand love. Some researchers have recently noted this, proposing that brand attachment can promote brand love (Santoso and Brahmana, 2019; Ghorbanzadeh and Rahehagh, 2020b; Thuy Tran, 2021). If consumers are satisfied with the brand experience, they will gradually become attached to the brand. After they become attached, they will have a deeper emotional connection with the brand based on brand love (Velicia Martín et al., 2020; Gilal et al., 2021; Shetty and Fitzsimmons, 2021). Therefore, the third hypothesis is proposed in this study:

H3. Brand attachment will positively affect brand love.

Brand loyalty is defined as “a firm commitment to continually repurchase or reuse a preferred product/service in the future, leading to repeated purchases of the same brand or same branded set, despite situational influences and marketing efforts that may lead to switching behavior” (Oliver, 1999, p. 34).

Consumers who identify with a particular brand and have positive feelings about it will become more loyal and actively promote it (Ýlter et al., 2016; Karjaluoto et al., 2016; Hsu and Chen, 2018). Maintaining online buyers in e-commerce platforms is viewed as a difficult task, and how to build loyalty among online shoppers is crucial for e-commerce platform companies (Yadav and Rahman, 2018; Al-Adwan and Al-Horani, 2019; Adwan et al., 2020). Brand loyalty should be higher under the influence of positive emotions, and satisfied consumers are more loyal to brands, and that brand love and loyalty are positively correlated (Christino et al., 2019; Fazli-Salehi et al., 2020; Putra and Keni, 2020). Therefore, the fourth hypothesis is proposed:

H4. Brand love will positively affect brand loyalty.

Similar to the ability of interpersonal attachment to predict relationships and commitment, brand attachment can also accurately predict consumer loyalty to brands, revealing consumer-brand interactions and predicting consumer commitment to brands and specific consumption behaviors (Park et al., 2010). Brand attachment drives brand loyalty, and a positive relationship exists (Rahmatiyah et al., 2017). Many studies have demonstrated a close relationship between brand attachment and brand loyalty (Bahri-Ammari et al., 2016; Jang, 2021; Kim and Ryu, 2021). Therefore, the fifth hypothesis is proposed in this study:

H5. Brand attachment will positively affect brand loyalty.

Repurchase intention is defined as the likelihood that a customer will engage in future repurchase behavior (Huete-Alcocer, 2017; Ramachandran and Balasubramanian, 2020). Brand loyalty refers to the tendency to buy the identical brand (Lee, 2020). Once consumers are loyal to a brand, they will buy the same product from that brand repeatedly (Shalehah et al., 2019; Prabowo et al., 2020). Currently, with the development of artificial intelligence technology, e-commerce platform companies have been able to explore customer brand perception and satisfaction through machine learning algorithms and big data (Hopkins, 2022; Klieštik et al., 2022b). E-commerce platform companies can also configure customer purchase intent through artificial neural network algorithms (Klieštik et al., 2022a; Nica et al., 2022). Many researchers have verified the positive relationship between brand loyalty and repurchase intention (Shafiee and Bazargan, 2018; Ali, 2019; Savila et al., 2019; Pranata and Permana, 2021; Laparojkit and Suttipun, 2022). Therefore, the sixth hypothesis is proposed:

H6. Brand loyalty will positively affect repurchase intentions.

Usually, the higher the level of attachment to a brand, the higher the level of affection for the brand (Shetty and Fitzsimmons, 2021). Emotional attachment to a brand leads to various positive emotions such as warm feelings, affection, and passion. Some studies have shown that positive relationships and brand attachment are prerequisites for love (Huang, 2017; Junaid et al., 2019). The existence of mediating effects has also been reported. Therefore, the seventh hypothesis is proposed:

H7. Brand love mediates the relationship between brand attachment and brand loyalty.

**FIGURE 1:**
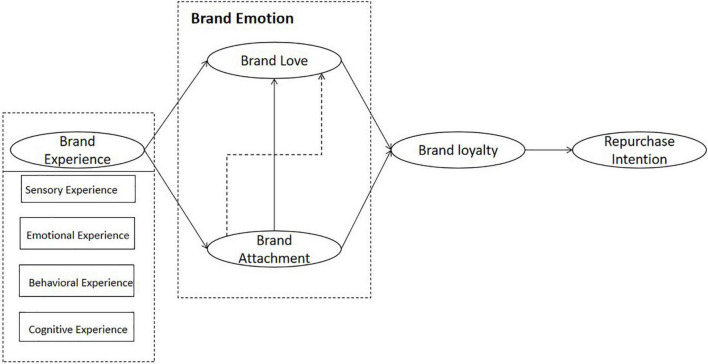
Research model. The dotted arrow is the mediating effect, and the solid arrows are the direct effect.

This study constructs the research model described above (see Figure 1).

## Methodology

### Questionnaire design

The measurement items for each construct in this study were adapted from previous studies, including brand experience (sensory, emotional, cognitive, and behavioral), brand attachment, brand love, brand loyalty, and repurchase intention. A five-point Likert scale was used in this study, described as follows, with 1 indicating strong disagreement and 5 indicating strong agreement. All these scales were initially written in English, and the questionnaire was finally translated into Chinese after consulting experts and making corrections.

In this study, the four-dimensional aspects of brand experience used the scales developed by Brakus et al. (2009) and Kim et al. (2018). For brand loyalty, the scales of Oliver (1999); Yoo and Donthu (2001), and Keller and Lehmann (2006) were used. For brand attachment, Thomson et al. (2005) and Kim et al. (2018) were used. For brand love, we used the scales of Carroll and Ahuvia (2006); Batra et al. (2012), Wallace et al. (2014), and Song et al. (2019). The scales of Oliver (1999); Lin and Lekhawipat (2014), Nikhashemi et al. (2019), and Chuah et al. (2022) were used for brand repurchase intention (as shown in the Appendix).

### Data collection and descriptive statistics of the sample

This study used an online cross-sectional survey. Before conducting the primary survey, this study conducted a preliminary test based on the data statistics website Alexa ranking and Avery Data APP ranking based on the weight calculation of the scale (TMO Group, 2022). Four well-known Chinese e-commerce brands were selected as the subjects of this study. They are Taobao, Pinduoduo, Douyin, and Jingdong. These platforms retain their customers through social media, mass media, and WOM to acquire them and develop customer-brand relationships, such as brand love and attachment, through brand experiences. Therefore, e-commerce platforms were an appropriate research context for this study.

This study tested the hypothesis by distributing questionnaires, initially screening out fifty people from China who had no experience in online shopping before the COVID-19 pandemic and started using online shopping after the outbreak as the survey respondents. Then removed inappropriate questions and established the final questionnaire. The last survey period was from July 1 to 20, 2021. Questionnaires were distributed and collected through the questionnaire company (Wenjuanxing). Finally, 328 questionnaires were collected, but 18 questionnaires with inappropriate or missing answers were removed, and 310 questionnaires were ultimately used in this study. The demographic characteristics of study population are shown in Table 1.

**TABLE 1:** Descriptive statistics.

Descriptive statistical indicators		Frequency (*n* = 310)	Percentage
Gender	Male	144	46.45%
	Female	166	53.55%
Age	18 years and younger	4	1.29%
	18–24 years old	92	29.68%
	25–30 years old	66	21.29%
	31–40 years old	55	17.74%
	40 years and older	93	30.00%
Education level	High School and below	69	22.26%
	College	69	22.26%
	Undergraduate	115	37.10%
	Masters and above	57	18.39%

## Analysis results and hypothesis testing

### Reliability and validity analysis of measurement models

First, the four dimensions of brand experience were tested in the SPSS 27.0 for their applicability. The KMO test value was 0.885, and the χ^2^ statistical value of Bartlett’s sphere test had a probability of significance of 0.000, indicating that the feasibility criteria of the PCA were fully met.

Second, the results shown in Tables 2, 3 were obtained through principal component analysis. The cumulative variance contribution of the four principal components extracted from Table 4 reached 72.6%, which indicates that they can adequately reflect the original data. The first principal component in Table 3 is summarized as cognitive experience. The second principal component is an emotional experience, the third principal component is a sensory experience, and the fourth principal component is behavioral experience.

**TABLE 2:** Rotated component matrix.

Indicators	Components 1	Indicators	Components 2	Indicators	Components 3	Indicators	Components 4
BE2	0.519	EE1	0.889	SE1	0.817	SE4	0.409
CE1	0.677	EE2	0.901	SE2	0.683	BE1	0.862
CE2	0.698	EE3	0.888	SE3	0.691	BE3	0.821
CE3	0.774	EE4	0.807	SE4	0.665	BE4	0.653
CE4	0.763			SE5	0.799		
CE5	0.793						

**TABLE 3:** Reliability and convergent validity analysis.

Items	UNSTD	S.E.	*t*-value	STD	SMC	CR	AVE	Cronbach’s alpha	CITC
SEN2	1.000			0.900	0.810	0.880	0.712	0.877	0.805
SEN3	1.054	0.059	17.858***	0.875	0.766				0.788
SEN4	0.842	0.055	15.210***	0.748	0.560				0.702
EMO1	1.000			0.952	0.906	0.959	0.886	0.958	0.920
EMO2	1.055	0.028	38.274***	0.968	0.937				0.930
EMO3	0.968	0.033	29.473***	0.902	0.814				0.883
BEH1	1.000			0.848	0.719	0.813	0.602	0.783	0.688
BEH3	1.064	0.090	11.773***	0.892	0.796				0.707
BEH4	0.777	0.083	9.321***	0.539	0.291				0.501
COG3	1.000			0.675	0.456	0.804	0.579	0.800	0.597
COG4	1.042	0.098	10.649***	0.784	0.615				0.664
COG5	1.206	0.114	10.601***	0.817	0.667				0.679
ATT1	1.000			0.711	0.506	0.762	0.517	0.761	0.589
ATT3	1.104	0.120	9.193***	0.762	0.581				0.616
ATT5	1.003	0.109	9.182***	0.682	0.465				0.572
LOVE2	1.000			0.865	0.748	0.869	0.690	0.868	0.771
LOVE4	0.995	0.064	15.56***	0.819	0.671				0.742
LOVE5	0.946	0.062	15.367***	0.806	0.650				0.733
LOY1	1.000			0.881	0.776	0.882	0.718	0.864	0.780
LOY2	1.077	0.055	19.471***	0.963	0.927				0.828
LOY3	0.903	0.065	13.801***	0.672	0.452				0.644
RI1	1.000			0.919	0.845	0.948	0.858	0.947	0.885
RI2	1.017	0.035	28.95***	0.943	0.889				0.901
RI3	1.020	0.038	27.005***	0.916	0.839				0.882

*p < 0.05, **p < 0.01, ***p < 0.001.

**TABLE 4:** Principal components analysis.

Component	Initial eigenvalue			% of variance (unrotated)			% of variance (rotated)		
	Total	% of variance	Cumulative %	Total	% of variance	Cumulative %	Total	% of variance	Cumulative %
1	8.305	46.137	46.137	8.305	46.137	46.137	3.624	20.132	20.132
2	2.046	11.369	57.506	2.046	11.369	57.506	3.620	20.114	40.245
3	1.480	8.225	65.731	1.480	8.225	65.731	3.245	18.028	58.273
4	1.237	6.870	72.600	1.237	6.870	72.600	2.579	14.327	72.600

Extraction method: Principal component analysis method.

In addition, with a Cronbach’s alpha coefficient of >0.7, the model was tested for reliability based on a corrected item-total correlation coefficient of 0.5, as proposed by Churchill (1979). The results are presented in Table 3. The eight dimensions of the model were sensory experience, emotional experience, behavioral experience, cognitive experience, brand attachment, brand love, brand loyalty, and repurchase intention. Cronbach’s alpha coefficients for all dimensions were greater than 0.7, and the CITC values were above 0.4, indicating that the questionnaire had high reliability.

A validation factor analysis was performed on the measurement model based on the reliability analysis, and the results are presented in Table 3.

The discriminant validity of these measures was demonstrated in the current study. Discriminant validity “assesses the extent to which measures of different concepts are different” (Bagozzi and Yi, 1988). It was validated by comparing the AVE of each measurement with the correlation of the responses associated with that measurement (Fornell and Larcker, 1981). This can also be proven by the fact that the square root of the AVE of each construct is higher than the correlation between its constructs. As shown in Table 5, the square root of each construct’s AVE (shown in bold) is mostly higher than its correlation estimate and the correlation between all other constructs, thus indicating a high level of validity.

**TABLE 5:** Discriminant validity.

	Mean	S.D.	(1)	(2)	(3)	(4)	(5)	(6)	(7)	(8)
Repurchase (1)	3.733	0.045	**0.926**							
Loyalty (2)	3.780	0.054	0.772	**0.847**						
Attachment (3)	3.463	0.053	0.792	0.772	**0.719**					
Love (4)	3.563	0.217	0.719	0.720	0.717	**0.831**				
Cognitive (5)	3.673	0.056	0.678	0.667	0.648	0.656	**0.761**			
Behavioral (6)	3.937	0.045	0.565	0.507	0.536	0.459	0.501	**0.776**		
Emotional (7)	3.417	0.056	0.513	0.510	0.502	0.518	0.454	0.333	**0.941**	
Sensory (8)	3.733	0.045	0.649	0.560	0.648	0.557	0.570	0.636	0.532	**0.844**
Reliability of construct			0.948	0.882	0.762	0.869	0.804	0.813	0.959	0.880

The Diagonal values are square root of the AVE, indicated in bold.

Measurement models can be evaluated by convergent validity and discriminant validity. According to Hair et al. (2010), convergent validity was evaluated by construct reliability and squared variance extraction. Discriminant validity was the correlation AVE between the construct and the mean-variance extracted. According to Table 3, the reliability of the construct meets the general criterion of 0.7 or higher, the mean-variance of all constructs is higher than 0.5, which is above the suggested threshold, and the measure is usually considered to have convergent validity.

In addition, Table 5 shows the extracted mean variances, indicating the ratio of the characteristic variance to the scale variance and the squared value of the correlation coefficient. To assess the discriminant validity, we checked whether the mean-variance extracted value exceeded the squared value of the correlation coefficient between the constructs. As seen in Table 5, the mean-variance extracted value is the squared value of the correlation coefficient between the constructs, which confirms the discriminant validity of the data collected by the model of this study.

Additionally, authors have also analyzed the multicollinearity regarding the correlation in this study. The results demonstrated a very low collinearity among the indicators, with VIF of all items ranging between 1 and 3, which is below the common cut off of 5 (Chin, 2010). So, there is no problem of multicollinearity in the model.

### Evaluation of the overall fitness of the equation model

Table 6 details the main fit indexes obtained from the structural model test. When compared with the fitness indexes’ recommended values, the fitted values of the fitness indexes fall within the recommended values, except for the AGFI value, which is very close to the recommended value of 0.9. It can be seen that the setting of this theoretical model is acceptable.

**TABLE 6:** The overall fit of the CFA model.

Model fit index	Recommended values	Fitted values
χ^2^	The smaller, the better	346.695
χ^2^/df	<3	1.548
RMR	<0.05	0.034
GFI	>0.9	0.920
AGFI	>0.9	0.892
RMSEA	<0.08	0.042
NFI	>0.9	0.951
RFI	>0.9	0.940
IFI	>0.9	0.987
TLI	>0.9	0.942
CFI	>0.9	0.979

### Results of testing the research hypothesis

**FIGURE 2:**
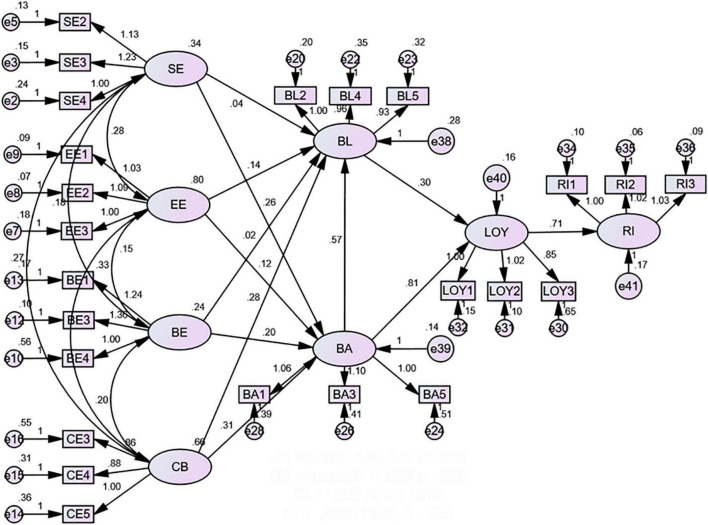
Structural model.

The structural relationships among the latent variables and the estimates of their standardized path coefficients, *t*-values, and hypothesis-testing results are presented in Table 7. It can be seen that all hypotheses passed the *T*-test, and most of the path coefficients were significant at the confidence level of α = 0.001. The actual model and the path coefficients obtained are shown in Figure 2.

**TABLE 7:** Hypothesis testing results.

Hypothesis	Estimate	*t*-value	Result
Brand sensory experience will positively impact brand love	0.044	0.377	Not supported
Brand emotional experience will positively impact brand love	0.139	2.506*	Supported
Brand behavioral experience will positively impact brand love	0.024	0.198	Not supported
Brand cognitive experience will positively impact brand love	0.279	3.208**	Supported
Brand sensory experience will positively impact brand attachment	0.257	2.933**	Supported
Brand emotional experience will positively impact brand attachment	0.117	2.807**	Supported
Brand behavioral experience will positively impact brand attachment	0.202	2.189*	Supported
Brand cognitive experience will positively impact brand attachment	0.305	5.151***	Supported
Brand attachment will positively impact brand love	0.568	3.808***	Supported
Brand love will have positively impact brand loyalty	0.297	4.050***	Supported
Brand attachment will positively impact brand loyalty	0.811	6.991***	Supported
Brand loyalty will positively impact repurchase intention	0.706	16.936***	Supported

*p < 0.05, **p < 0.01, ***p < 0.001.

### Analysis of the mediation effect of brand love

The tests for significant mediating effects include the sequential test, MCMC method, Sobel test, and bootstrap test. Among these, the bootstrap test is the most accurate test of the mediating effect. In this study, AMOS structural equation modeling software was used to test the mediating effect of brand love based on the bootstrap method proposed by Hayes and Preacher (2013). Considering that the bootstrap method in the structural equation modeling software calculates the total mediating effect and cannot test the mediating effect of specific parts, the PRODCLIN2 program of MacKinnon and Luecken (2008) was also used in this study to test the mediating effect of specific paths of the model. The coefficients and standard errors of the unstandardized paths from the independent variable to the mediating variable and the mediating variable to the dependent variable, and the standardized total direct effects were estimated from the parameters obtained from the AMOS software. If the 95% confidence interval of the calculated results did not include 0 after the execution of the procedure, the mediating effect of the path was significant.

First, the total mediating effect was tested for significance and degree of the mediating effect. In Table 8, the point estimates and standard errors of the total mediated effects are calculated using the bootstrap method for the path parameter estimates. *Z*-values are point estimates/standard errors, and absolute values of *Z*-values greater than 1.96 indicate significance. The data showed that the total direct effect of brand love was 0.663, and the 95% confidence interval of Bias-corrected and 95% confidence interval of percentile did not contain 0. This indicates that brand love has a significant mediating effect on brand attachment and loyalty. In addition, the total direct effect was 0.312, the 95% confidence interval of bias-corrected and the 95% confidence interval of percentile did not contain 0, indicating that the indirect effect was significant, and the type of mediation effect of brand love on brand attachment and loyalty was partially mediated.

**TABLE 8:** Mediating effects.

Variables	Estimate			Bootstrapping				Mackinnon	
		Product of coefficients		Bias-corrected		Percentile		PRODCLIN2	
				95% CI		95% CI		95% CI	
		
		SE	*Z*	Lower	Upper	Lower	Upper	Lower	Upper
		Total effects							
BA→LOY	0.975	0.101	9.653	0.800	1.182	0.800	1.178	0.783	1.171
		Direct effects							
BA→LOY	0.663	0.075	8.840	0.178	0.468	0.162	0.460	0.143	0.529
		Indirect effects							
BA→LOY	0.312	0.123	2.537	0.440	0.920	0.450	0.929	0.442	0.884

1000 bootstrap samples.

The mediating effects of the specific mediating factors were then tested for significance. The partial mediation effect test results in Table 8 show that the 95% confidence interval of Mackinnon PRODCLIN2 does not contain 0, indicating that the mediation effect of this path is significant and verifying H7. According to the above analysis, brand love is partially mediated by brand attachment and loyalty.

## Discussion

### Findings

This study was conducted on new e-commerce users during the COVID-19 pandemic. Additionally, the structural relationships between brand experience, brand attachment, brand love, brand loyalty, and repurchase intention were investigated. The results of the structural equation modeling showed that, first, the four dimensions of brand experience had a significant positive relationship with brand emotion, among which brand perception experience had the most significant impact on consumer brand emotion. Second, the influence of brand emotion on brand loyalty is positive and significant, and brand attachment had a more decisive effect than brand love on brand loyalty. To meet the needs of customers, e-commerce platform companies are primarily focused on attachment rather than love. Finally, brand loyalty positively affects brand repurchase intention; the higher the brand loyalty, the stronger the repurchase intention.

### Theoretical implications

This research makes contributions to the literature in three ways. First, this study focused on customers who had no experience in online shopping prior to the COVID-19 pandemic and turned to e-commerce during the COVID-19 pandemic. No previous studies have focused on this population; therefore, this study fills a gap in this area.

Second, the paper subdivides and discusses the brand experience by dividing it into four dimensions: sensory, emotional, action, and cognitive. A majority of previous studies were conducted in the Western context, and there are few studies of subdivided dimensions in the East Asian cultural circle, especially in China. This study contributes to branding literature by demonstrating that cognitive experience is the most important of these four dimensions. In addition, many prior studies have considered brand loyalty as an outcome (Yadav and Rahman, 2018; Al-Adwan and Al-Horani, 2019; Adwan et al., 2020), and we investigate the relationship between brand loyalty and repurchase intention based on the prior studies.

Finally, this study also examines the mediating effect of brand love on brand attachment and loyalty, making the customer’s consumption process clearer and more hierarchical. The triadic theory (Sternberg, 1986) in consumer psychology has been widely recognized (Shimp and Madden, 1988; Ahuvia, 2005; Amani, 2022), but there is still a lack of research using emotional factors as mediating variables. This research is conducted to enhance the existing literature by examining the different effects of the antecedents in the research model. Although research on brand love has been increasing recently, few studies have used brand love as a mediating effect to verify the relationship between brand attachment and brand loyalty. In this research, brand love was used as a mediating variable, and future studies can use this idea to explore more mediating variables of emotion.

### Practical implications

This study contributes to the managerial implications in three aspects. First, this study differs from previous ones in that it utilizes Chinese consumers who are new to online shopping during the COVID-19 pandemic outbreak as the research object. The authors tested the relationship between brand attachment and brand love by subdividing brand experience. The results show that at the brand experience level, cognitive experience has the most significant impact on brand attachment and brand love. It was verified that brand experience is the antecedent of brand attachment and brand love. These results are somewhat different from those of previous studies involving brand experience. Specifically, brand experience in this study had the highest brand cognitive experience of brand attachment (*B* = 0.305, *t*-value = 5.151, *p* < 0.001), and the results of some prior studies were similar to those of this study (Lee and Kim, 2020; Rodrigues and Brandão, 2020; Nikolinakou et al., 2021). However, there are also some studies with discrepant results; for example, Huang (2017) argues that sensory experience has the highest impact on brand attachment. Some studies also consider behavioral experience to have the highest impact on brand attachment (Cho, 2020; Shang et al., 2020). Brand experience plays a crucial role in maintaining the relationship between the customers and the e-commerce platform operators. The cognitive experience of the brand should be enhanced to increase customer attachment and love for a brand. In the context of growing competition on e-commerce platforms, cognitive experience plays an essential role in providing new value to its customers. To increase customers’ ways of access to cognitive experience, a good experience will naturally be widely recognized by customers and eventually form a positive emotion toward the brand. Therefore, e-commerce platform operators should offer newer and more popular products than other platforms, and provide differentiation strategies that make people feel curious and surprised in terms of cognition, provide customers with information and reviews about the products, reduce the uncertainty and risk of online shopping, and thus make customers feel optimistic about the brand. Through these positive brand experiences, customers can develop trust in a particular online platform, which will eventually go beyond brand attachment and love to brand loyalty. Therefore, e-commerce platform operators should provide customers with the opportunity to experience a variety of content.

Second, the results show that brand attachment has a positive effect on brand love, and brand attachment and love have a positive effect on brand loyalty. Thus, brand attachment is the antecedent of brand love, and in this regard, the results of this study are the same as those of previous studies (Gómez-Suárez, 2019; Ghorbanzadeh and Rahehagh, 2020a; Shetty and Fitzsimmons, 2021). And brand attachment and brand love are essential factors influencing brand loyalty; these results are consistent with those of previous studies (Santoso and Brahmana, 2019; Hwang et al., 2021; Rahman et al., 2021). To increase brand love and loyalty, e-commerce platform companies must strengthen their customers’ brand attachment. Thus, e-commerce platform companies should differentiate their brands from other brands to increase customer attachment to the brand (Ghorbanzadeh and Rahehagh, 2020b). For example, e-commerce platform operators can develop communication strategies, such as social media marketing advertising, to give customers a favorable impression of the brands.

Third, the results show that brand loyalty has a positive effect on repurchase intention. This means that brand loyalty is an antecedent to repurchase intention. Based on these results, it is necessary to improve brand loyalty to enhance customers’ repurchase intentions. Generally speaking, brand loyalty plays a vital role in maintaining the relationships between customers and operators. If customers are loyal to a particular platform, their propensity to repurchase will increase. Loyal customers to a specific seller may have purchasing power ten times stronger than ordinary customers (Anderson and Srinivasan, 2003). E-commerce platform companies should introduce artificial intelligence into more aspects of online shopping to reduce consumer perceptions of brand risk through machine learning agents and self-service technologies, which will also improve customer perceptions of interaction efficiency (Hopkins, 2022; Klieštik et al., 2022b; Nica et al., 2022). Digital technology has not only reinvented online shopping but can also help increase brand loyalty (Klieštik et al., 2022a). Although there is uncertainty regarding the use of AI technology by e-commerce platform operators, the possibility of rapid technological development is high. Therefore, e-commerce platform companies should use artificial intelligence to ensure profitability in the short and long term.

### Limitations and future research

Despite the exciting results of this research, this study has some limitations. First, the respondents in the survey were solely Chinese. Future studies could be replicated in other countries to further generalize the findings and perform a comparative analysis of Eastern versus Western consumption perceptions. Second, moderating variables were not included in this study, and the appropriate inclusion of income, gender, and age as moderating variables for future studies should be considered. Third, the direct relationship between brand experience and repurchase intention has not been explored, but several scholars have conducted research in this area (Lãzãroiu et al., 2017; Andronie et al., 2021; Barbu et al., 2021; Vinerean et al., 2022). Therefore, future research can further include this direct relationship. Finally, this study was conducted on people who did not purchase online before the COVID-19 pandemic outbreak, but started purchasing online for the first time after the outbreak. However, no comparison was made between customers who had experience in online shopping before the COVID-19 pandemic outbreak and those who started online shopping only after the outbreak. Therefore, a comparative analysis of these two groups is necessary for future studies.

## Conclusion

Due to the pandemic, not only general consumers but also workers with high purchasing power have been forced to work from home instead of going to work for safety and to prevent the spread of the pandemic. As a result, with spending more time at home, people who had not previously thought about online buying are more likely to move to online shopping for various reasons. There were many negative factors caused by the pandemic, but in the case of online companies, there may be an opportunity. In order to take advantage of such opportunities, it is vital for companies to implement active sales strategies in order to turn online purchasers into loyal customers and to keep them repurchasing.

Unlike prior studies conducted before the pandemic, this study analyzed four dimensions of brand experience in the Chinese context. The results show that from the perspective of brand experience, cognitive experience has the most significant impact on brand attachment and brand love. To build brand attachment and brand love, e-commerce platform companies must provide customers with new cognitive experiences to arouse their curiosity and interest, and increase the sources of product reviews. From the perspective of brand experience, building brand attachment and love can increase consumer loyalty to the brand and ultimately increase repurchase intention. With the recent rapid development of artificial intelligence technology, online shopping platforms must introduce this technology. For e-commerce platform companies operating a large number of products, companies should reflect consumers’ interests and preferences and provide customized recommendation services through artificial intelligence, which increases customers’ attention to new products and their understanding of product-related information, and helps increase the repurchase intention of consumers as well as the platform’s revenue.

## Data availability statement

The original contributions presented in this study are included in the article/supplementary material, further inquiries can be directed to the corresponding author.

## Ethics statement

Ethical review and approval was not required for the study on human participants in accordance with the local legislation and institutional requirements. Written informed consent for participation was not required for this study in accordance with the national legislation and the institutional requirements.

## Appendix

**Table:**

Dimensions	Items
Sensory experience	SE1 The e-commerce platform impressed my visual and other senses.
	SE2 I think the e-commerce platform is interesting in a sensory way.
	SE3 The e-commerce platform appealed to my senses.
	SE4 I would like to see the products of the e-commerce platform with my own eyes.
	SE5 I would like to see examples of products using this e-commerce platform.
Emotional experience	EE1 The e-commerce platform evokes my sensibility.
	EE2 The products of this e-commerce platform touched my feelings and emotions.
	EE3 The products of this e-commerce platform can make me feel happy.
	EE4 I want to get a pleasant mood from the products of this e-commerce platform.
Behavioral experience	BE1 Wants to participate in activities related to the products of this e-commerce platform.
	BE2 I would like to use the products of the e-commerce platform myself.
	BE3 The products of this e-commerce platform offer a new way of life.
	BE4 I want to apply the products of the e-commerce platform to my lifestyle.
Cognitive experience	CE1 The e-commerce platform piqued my curiosity and facilitated problem-solving.
	CE2 Wants to know professional information about e-commerce platform products.
	CE3 Wants to know the user’s evaluation of the e-commerce platform products.
	CE4 The products of this e-commerce platform provided me with the necessary information.
	CE5 The products of this e-commerce platform draw attention to new information.
Brand loyalty	LOY1 I will be talking to people about this e-commerce platform.
	LOY2 I would recommend this e-commerce platform to others.
	LOY3 I am willing to pay a higher price for a product from this e-commerce platform.
	LOY4 If the product I want to buy is out of stock on the relevant e-commerce platform, I will wait for it to be restocked.
Brand attachment	BA1 The e-commerce platform is very enthusiastic.
	BA2 The e-commerce platform evokes positive feelings in me.
	BA3 The e-commerce platform is very close to me.
	BA4 I love the e-commerce platform.
	BA5 I can feel the charm of the e-commerce platform
Brand love	BL1 I think the e-commerce platform is a great brand.
	BL2 The e-commerce platform makes me happy.
	BL3 Negative sentiment toward the e-commerce platform (-).
	BL4 The e-commerce platform makes me feel happy.
	BL5 I love the e-commerce platform.
	BL6 I like the e-commerce platform.
Repurchase intention	RI1 The products of this e-commerce platform are worth buying.
	RI2 I am interested in purchasing products from this e-commerce platform in the future.
	RI3 I will give priority to this e-commerce platform for my next purchase.
	RI4 I will continue to buy products from this e-commerce platform in the future.
